# Biotinidase Deficiency: Report of a Tunisian Case With Neuromyelitis Optica-Like Presentation and Review of the Literature

**DOI:** 10.1155/crnm/7003370

**Published:** 2025-03-25

**Authors:** Abir Zioudi, Hanene Benrhouma, Maha Jamoussi, Thouraya Ben Younes, Zouhour Miladi, Hedia Klaa, Sonia Nagi, Brahim Tabarki, Ilhem Ben Youssef Turki, Ichraf Kraoua

**Affiliations:** ^1^LR18SP04 and Department of Pediatric Neurology, National Institute of Neurology Mongi-Ben Hamida, Tunis El Manar University, Tunis, Tunisia; ^2^LR18SP04 and Neuroradiology Department, National Institute of Neurology Mongi-Ben Hamida, Tunis El Manar University, Tunis, Tunisia; ^3^Department of Pediatric Neurology, Prince Sultan Military Medical City, Riyadh, Saudi Arabia

## Abstract

Biotinidase deficiency is a rare treatable metabolic disorder caused by biallelic mutations in the *BTD* gene. In the absence of neonatal screening and treatment, affected children develop typically optic atrophy, hypotonia, early onset seizures, developmental delay, and cutaneous manifestations. Some patients may have atypical presentations mimicking a demyelinating disorder of the central nervous system. We report on the first genetically confirmed Tunisian patient with biotinidase deficiency who presented initially with cutaneous manifestations misdiagnosed as dermatophytosis and subsequently with an opticospinal syndrome leading to the diagnosis of seronegative neuromyelitis optica spectrum disorder that was dramatically improved under biotin. We carry on a review of the literature of the previously reported pediatric cases with an opticospinal syndrome revealing biotinidase deficiency.

## 1. Introduction

Biotinidase deficiency (BD) is a rare autosomal recessive neurometabolic disorder associated with mutations in the *BTD* gene. It is caused by the inability to recycle biotin, an essential B vitamin that acts as a coenzyme of four carboxylation enzymes: propionyl–CoA carboxylase, β-methylcrotonyl–CoA carboxylase, acetyl–CoA carboxylase, and pyruvate carboxylase. These carboxylases are involved in amino acid catabolism, fatty acid synthesis, and gluconeogenesis [1]. The clinical presentation includes neurological, neurosensory, and cutaneous manifestations of variable severity. Some patients may present with early life-threatening infantile encephalopathy, while others remain asymptomatic [1]. A few cases of optic neuropathy and myelitis resembling neuromyelitis optica (NMO) spectrum disorders (NMOSD) have been reported [1, 2]. Diagnosis may be delayed, compromising the prognosis.

In this report, we present the first genetically confirmed Tunisian case of BD with an NMOSD-like presentation and discuss the clinical and paraclinical characteristics of this patient, along with a review of the literature.

## 2. Case Report

The patient is a 7-year-old girl born from a second-degree consanguineous marriage, with no remarkable family history. The pregnancy and delivery were uneventful. She had a history of neonatal jaundice secondary to a maternal–fetal infection which was treated with antibiotics and phototherapy. She had normal psychomotor development, and she excelled at school.

The disease history dates back to the age of 6 years and 9 months when she developed itchy and crusty papular erythematous lesions on her back, which gradually disappeared within 2 weeks. Concomitantly, she presented with bilateral blepharitis, which was treated with unspecified eye drops. Three months later, she began experiencing progressive hair loss affecting the entire scalp. A mycological examination of the hair revealed the presence of *Microsporum canis*, leading to the diagnosis of dermatophytosis. She was treated with antifungal therapy, but her condition did not significantly improve. After a month, she developed gradual visual loss, concurrent with the recurrence of an episode of blepharitis that was considered to be the cause of the visual disorder. Subsequently, she started to experience progressive weakness in her lower limbs, leading to gait disturbances and frequent falls 1 month later. During this time, her skin lesions reappeared and spread to her right lower limb. Otherwise, she did not exhibit sphincter control disorders or sensory disturbances. Due to these concerning symptoms, she was referred to our department for further investigations.

**Figure 1:**
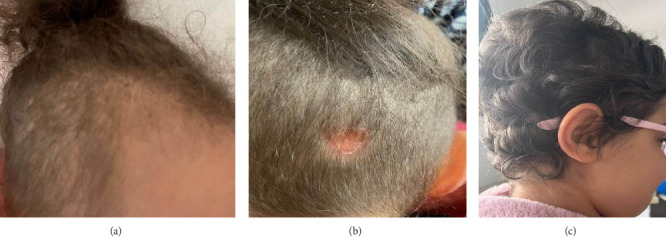
Hair involvement in our patient and evolution under biotin treatment. (a) Diffuse alopecia. (b) A well-limited dry and scaly occipital patch covered with very short hair suggestive of ringworm. (c) Hair regrowth after treatment with biotin.

Upon physical examination, we observed spastic tetraparesis predominantly in the lower limbs with a spastic gait as well as bilateral decreased visual acuity. She also displayed diffuse alopecia (Figure 1(a)) and a well-defined dry and scaly occipital patch covered with very short hair, suggestive of ringworm (Figure 1(b)). Determining visual acuity was challenging as the child was not cooperating, but it seemed significantly impaired. The eye fundus, however, appeared normal. Visual evoked potentials (VEPs) indicated bilateral increased P100 wave latency.

**Figure 2:**
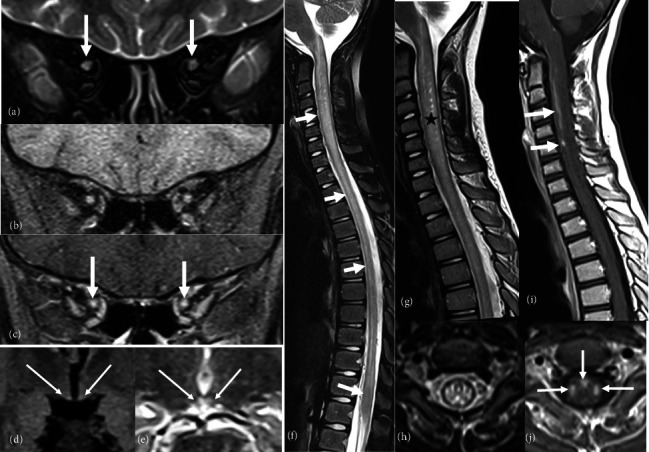
Radiological findings. (a–c) Coronal T2 (a), T1 with fat saturation (b) and T1 with fat saturation after gadolinium administration (c) weighted images on optic nerves. Bilateral increased T2 signal in the intra orbital optic nerves with enhancement (arrows). (d, e) Coronal fluid-attenuated inversion recovery (d) and T2 (e) weighted images. Bilateral hyperintensities involving the mammillary bodies (thin arrows). (f–h) Sagittal (f, g) T2-weighted images on the spinal cord. Hyperintense extensive lesion of the spinal cord from the bulbomedullary junction to the medullary cone (short arrows) with cervical spinal cord swelling (asterisk). (h) Axial T2-weighted image on the spinal cord showing the bilateral and symmetrical aspect of the hyperintensities which are selective, involving anterior, lateral and posterior columns. (i, j) Sagittal (i) and axial (j) T1-weighted images after gadolinium administration. Lateral and anterior column enhancement (arrows).

A brain and spinal magnetic resonance imaging (MRI) was performed, revealing extensive myelitis affecting the entire spinal cord, extending to the conus medullaris and the medulla oblongata, with enhancement areas after contrast injection. The spinal cord appeared swollen. Brain MRI showed bilateral hyperintensities involving the mammillary bodies. In addition, orbital MRI showed T2 hyperintensities and enhancement of the optic nerves in their intraorbital segments (Figure 2).

Considering the clinical presentation of optic neuritis and longitudinally extensive myelitis, we considered a diagnosis of NMO or myelin oligodendrocyte glycoprotein (MOG) antibody disease (MOGAD). MOGAD was more suspected due to the mild clinical presentation, conus medullaris involvement, and anterior bilateral optic neuritis. Consequently, she was put on a 5-day corticosteroid bolus. On the second day of treatment, we noted a worsening of the deficit in the lower limbs, making walking impossible without assistance. Intravenous immunoglobulin therapy was added, but unfortunately, it did not yield improvement.

A lumbar puncture with cerebrospinal fluid (CSF) analysis was performed, revealing normal cellularity and proteinorachia. However, lactate level was elevated at 5.41 mmol/L (normal values < 2 mmol/L). Assays for anti-aquaporin 4 (AQP4) and anti-MOG antibodies returned negative results. The diagnosis of BD was considered; hence, the patient initiated biotin therapy at a dosage of 15 mg/day.

**Figure 3:**
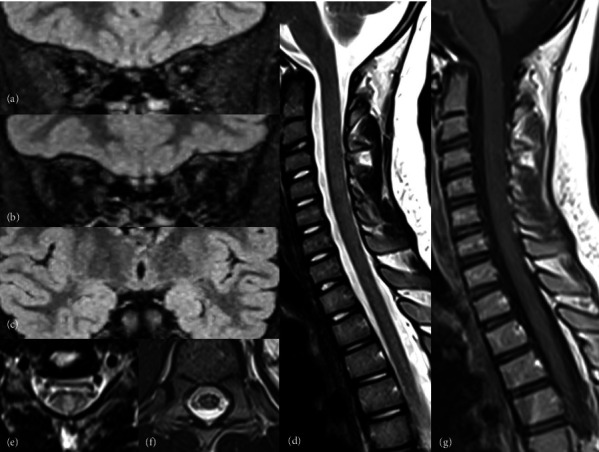
Control cerebrospinal and orbital MRI after biotin supplementation. (a–c) Coronal fluid–attenuated inversion recovery weighted images showing a normal signal on the optic nerves and mamillary bodies. Sagittal (d) and axial (e, f) T2 weighted images on the spinal cord. Persistence of a mild hyperintense signal of the cervical posterior tract. (g) Sagittal T1 weighted image after gadolinium administration. Disappearance of the medullary enhancement.

Her symptoms progressively and significantly improved. Alopecia was the first symptom to recover, with full hair regrowth (Figure 1(c)). On the motor level, the child resumed normal walking after 4 months of treatment. Visual acuity improved to 6/10 on the right and 3/10 on the left after 3 months of treatment. Nonetheless, optical coherence tomography (OCT) revealed bilateral temporal optic atrophy. A follow-up ophthalmological examination at 7 months of treatment showed a visual acuity of 8/10 on the right and 6/10 on the left eye. However, the control OCT and VEP showed the same abnormalities. Radiologically, follow-up cerebrospinal and orbital MRI demonstrated marked improvement (Figure 3). Auditory evoked potentials were performed and returned normal results.

Genetic testing identified a homozygous missense variant: c.1553G > T p.(Arg518Leu) in the *BTD* gene. All in silico tools utilized predicted this variant to be deleterious, confirming the diagnosis of BD.

## 3. Discussion

We report the first genetically confirmed case of BD in Tunisia, which presented as NMOSD. BD is a rare disorder with an estimated incidence of 1:40,000–1:60,000 births worldwide [1]. The prevalence is higher in countries with high consanguinity rates, such as Turkey and Saudi Arabia [1]. Data about its frequency in Maghreb countries are lacking. This disease is most likely underestimated, due to the lack of newborn screening, already implemented in many other countries, and the difficulty to perform genetic or biochemical confirmation.

In our case, this condition was attributed to a variant previously classified as a variant of undetermined significance (VUS) in ClinVar (variation ID: 439035). Notably, other missense variants within the same codon, namely, p.(Arg518Cys), p.(Arg518His), and p.(Arg518Ser), have been reported in individuals displaying BD–related phenotypes [3–11]. This suggests that the affected amino acid plays a significant functional role. Furthermore, in silico tools have predicted this variant to be deleterious. Given the clinical presentation and the substantial improvement observed with biotin treatment, the pathogenicity of this variant was considered valid, although the measurement of enzyme activity could not be performed due to the unavailability of this test in Tunisia.

Cases involving optic neuropathy and myelitis occurring either simultaneously or sequentially have been documented. The age of onset typically occurs during childhood, adolescence, or even adulthood, which contrasts with the common presentations that typically manifest during infancy or the neonatal period. These more typical presentations are associated with severe symptoms such as seizures, hypotonia, developmental delay, intellectual disability, ataxia, deafness, optic atrophy, feeding difficulties, respiratory problems, impaired consciousness, as well as cutaneous manifestations, including eczematous rash and alopecia [1, 2]. It is worth noting that skin involvement may be absent, especially in later presentations, which can contribute to delayed diagnosis [2, 12]. In our case, the disease initially presented with cutaneous symptoms; however, diagnosis was delayed due to the nonspecific nature of the findings and their association with dermatophytosis. Recurrent infections, including fungal infections, have been reported in individuals with BD, particularly candidiasis [1, 13–16]. The hypothesis explaining this increased susceptibility to infections points towards immunologic dysfunction [13, 14, 17]. Blepharitis has also been reported among ophthalmological manifestations and should raise suspicion of the diagnosis, especially when it recurs and is associated with other skin manifestations [14, 18, 19].

Opticospinal syndrome has been reported in 14 pediatric patients, and their demographic, clinical, and paraclinical data are summarized in Table 1 [4, 19–31].

Optic involvement typically preceded the onset of motor symptoms or was discovered during routine examinations or detected through VEP or MRI. Visual loss often exhibited a progressive course, although instances of sudden onset or acute deterioration have been documented [28]. Ophthalmological examinations commonly revealed optic atrophy. Nerve fiber layer loss, primarily affecting the temporal quadrant, was observed in a 19-year-old patient [32], and altered VEP results were prevalent among the majority of patients. MRI findings included T2 hypersignal, thickening, or enhancement of the optic nerves, with involvement extending to the intraorbital segments [28, 29]. Chiasmatic involvement was also reported [26, 28].

As for myelopathy, clinical manifestations frequently presented as motor difficulties and limb weakness, with less common occurrences of sphincter or sensory disorders. The onset of symptoms is typically progressive or subacute, often accompanied by evident spasticity upon physical examination. MRI examinations consistently revealed extensive myelitis, typically affecting the cervical or the entire spinal cord, extending up to the conus medullaris, and manifesting as spinal cord swelling and patchy enhancement [25, 29]. These features strongly suggest an inflammatory demyelinating disorder of the central nervous system (CNS), which can lead to misdiagnosis, as in our case. Literature reports also indicate involvement of the anterior, lateral, and posterior columns [27]. Furthermore, a few cases have described radiologically asymptomatic involvement of the area postrema [29].

Lactate levels in CSF or serum were elevated in the majority of BD cases. Notably, high lactatorachia has been reported in cases of NMO, with levels ranging between 2.1 and 6.8 mmol/L, although significant elevation remains relatively rare, with a median of 2.9 mmol/L [33]. In addition, some patients with MOGAD exhibited a moderate increase in lactatorachia, with levels never exceeding 3 mmol/L in pediatric patients [34]. Therefore, it is recommended to assess lactate levels in both blood and CSF in individuals with NMO-like presentations, especially when atypical findings are present.

Clinical and/or radiological worsening despite corticosteroid treatment or their ineffectiveness has been documented in several cases. In some instances, patients exhibited only partial improvement when treated with steroids, either alone or in combination with immunotherapy. In cases of such inadequate response, it is advisable to reconsider the diagnosis of NMO or MOGAD and consider alternative diagnoses, including BD.

Treatment with biotin should be initiated as early as possible to prevent further deterioration and the development of neurological or neurosensory sequelae. Dosages of 10–20 mg per day have proven sufficient to reverse the manifestations, and treatment should be continued for the patient's lifetime. The prognosis under treatment has been favorable in all reported cases in the literature, as in our patients. Unsurprisingly, the best outcomes are those of patients treated in the presymptomatic phase, which emphasizes the importance of neonatal screening. Pending its implementation in the Maghreb countries, at-risk family screening allows early diagnosis and better prognosis in affected patients. Where applicable, physicians must be made aware of the different atypical presentations in order to recognize them since they benefit from a simple treatment which dramatically transforms the prognosis.

## 4. Conclusion

BD is a rare neurometabolic disorder that can mimic demyelinating inflammatory CNS disorders such as multiple sclerosis, NMO, or MOGAD. It is crucial to consider this diagnosis in children, especially when accompanied by cutaneous manifestations such as alopecia, eczematous dermatitis, or blepharitis or when atypical clinical, biochemical, or evolutionary findings are present. This is because BD is one of the few treatable genetic disorders, and with biotin supplementation, it offers an excellent prognosis.

## Figures and Tables

**Table 1:** Demographic, clinical, and paraclinical data of all pediatric patients with biotinidase deficiency and opticospinal involvement.

	Our case	Patient 1	Patient 2	Patient 3	Patient 4	Patient 5	Patient 6	Patient 7	Patient 8	Patient 9	Patient 10	Patient 11	Patient 12	Patient 13	Patient 14
Reference		[20]	[21]	[22]	[23]	[24]	[19]	[25]	[26]	[27]	[28]	[4]	[29]	[29]	[30]
Origin	Tunisia	Poland	England	Laos	Cyprus	England	China	Pakistan	India	India	Turkey	Caucasian⁣^∗^	Australia	Australia	Iran
Gender	F	M	F	M	F	M	M	M	M	M	M	F	F	F	F
Consanguinity	+	−	−	−	+	−	NM	+	−	NM	−	−	−	NM	+
Psychomotor development	Normal	Normal	Normal	NM	Normal	NM	Normal	NM	Normal	NM	Normal	Normal	Delayed	Language delay	Normal
Age at onset (y)	6.8	10.5	5	10	1.3	2	5	1.8	5	14	11	4	2.2	0.5	2,8
Inaugural manifestations	Skin lesions	Acute visual loss	Progressive visual loss	Progressive visual loss	Seizures + skin lesions	Seizures, ataxia, and alopecia	Blepharoconjunctivitis and visual loss	Progressive motor weakness and ataxia	Progressive quadriparesis and respiratory disturbances	Progressive visual loss and difficulty in walking	Progressive visual loss	Acute tetraparesis, with urinary retention	Intermittent ataxia, torticollis, and irritability	Swallowing and respiratory problems	Gait disturbances + gradual visual loss
Clinical symptoms															
Visual loss	+	+	+	+	+ (on examination)	+	+	−	−	+	+	+	−	−	+
Gait disorders	+	−	+	+	+	+	+	NM	+	+	+	+	+	−	+
Weakness of limbs	+	+	−	+	+	+	+	+	+	+	+	+	−	−	+
Sphincter disorders	−	−	−	−	+	−	−	+	−	+	−	+	−	−	−
Cutaneous involvement	Dermatitis, alopecia, and blepharitis	−	−	Periorbital dermatitis	Skin rash	Skin rash	Alopecia, perianus, and periorofacial macular rash	Friable and sparse hair	−	−	−	Mild eczema	−	−	−
Other	−	Fatigability and chronic conjunctivitis	−	−	Hearing loss, respiratory difficulty, and dysarthria	Fatigue, diplopia, respiratory difficulty, ndhearing loss	Intermittent vomiting tremor, lethargy, anorexia	−	Swallowing difficulties	−	Diplopia	Respiratory distress	Tachypnea	−	Recurrent upper respiratory tract infections
Neurological examination	Spastic tetraparesis	Signs of upper and lower MN involvement, bilateral ptosis, and reduction of vibration sense in LL	Spastic paraparesis	Spastic tetraparesis and rigidity of the paraspinal muscles	Motor deficit in upper and LL, hypesthesia below T12, reduction of vibration sense in LL, and visual loss	Spastic paraparesis	Spastic paraparesis and nystagmus	Spastic paraparesis	Hypophonic speech, bulbar palsy, and spastic quadriplegia	Spastic paraparesis	Horizontal nystagmus and motor deficit in upper limbs	Tetraplegia and blindness	Torticollis, hypotonia, and ataxic gait	NM	Flaccid quadriplegia
Visual acuity	NM	20/200: RE 20/400: LE	0,5/60 in both eyes	20/300 in both eyes	NM	NM	20/200 in both eyes	NM	−	Decreased	5/100 RE 10/100 LE	NM	NM	−	NM
Eye fundus	Normal	Optic atrophy	Optic atrophy	Optic atrophy	Optic atrophy	Optic atrophy	Optic atrophy	Optic disc pallor	NM	Optic atrophy	Optic disc pallor	NM	NM	NM	Optic atrophy
VEP	Increased P100 wave latency	Diminished amplitudes and prolonged latencies	Absent	RE: Extinguished pattern, LE: trace response	Prolonged P100 wave latency	NM	NM	Bilateral marked postretinal dysfunction	NM	NM	Bilateral partial conduction defect	NM	NM	NM	NM
CNS imaging															
Spinal MRI	Extensive myelitis to the conus and medulla	Normal	NM	Normal	NM	Abnormal (details not specified)	Extensive cervical myelitis	Extensive myelitis to the conus and inferior brainstem	Extensive myelitis from the medulla to the conus	Extensive myelitis affecting the anterior, lateral, and posterior columns of the entire SC	Cervical extensive myelitis with contrast enhancement	Extensive myelitis from the CMJ to the level of T10 affecting the posterior and lateral ropes	Extensive myelitis from the medulla to the conus	Extensive myelitis from the CMJ to the thoracic cord	Extensive cervical myelitis from C2 to C5 with mild cord edema
Signal anomalies in brain MRI	+ Dorsal medulla involvement (area postrema)	No	No	No	NM		No	+ Medial thalamus, tectum, PGM, dorsal pons, and medulla	+ Septum pellucidum, CC, fornix, medial thalamus, dorsal midbrain, PGM, dorsal pons, and medulla	NM	+ Tectal plate	+ Medulla and mammillary bodies	+ Mammillary bodies, dorsal medulla, and area postrema	+ Mammillary bodies, PGM, and area postrema	NO
Orbital MRI	T2 hyperintensities + enhancement of the ON	NM	NM	Not performed	NM		Thick optic nerves	NM	Signal abnormalities of the optic tracts and chiasm	NM	T2 hyperintensities of the intraorbital segments of the ON and chiasm	T2 hyperintensities of the ON	Bilateral optic neuritis	Bilateral optic neuritis	NM
Lactate															
In serum (mmol/L)	−	NM		NM	Slightly elevated	NM	Slightly elevated	↑ 2.7	NM	NM	Normal	↑ 5.1	Normal	↑ 2.8	↑ 2.8
In CSF (mmol/L)	↑	NM	↑ 7.39	NM	NM	NM	NM	↑ 5.9	↑ 8,73	NM	NM	↑ 8.54	↑ 7.0	NM	↑ 6,8
Mutation	c.1553G > T p. (Arg518Leu)	c.643C > T p. (L215F)	NM	c.1369G > A p. (V457M)	c.1612C > T p. (R538C)	G98:d7i3 and c.643C > T p. (L215F)	−	−	c.133C > T p. (H447Y)		c.98_104delinsTCC p. (C33Ffs∗36)	c.643C > T p. (L215F) + c.1612C > T p. (R538C)	c.1612C > T p. (R538C)	c.100G > A p. (Gly34Ser)	c.838A > C (p.Asn280His)
Treatment with steroids	+	−	−	−	−	−	+	+	+	−	+	+	+	−	NM
Evolution under steroids	Worsening	−	−	−	−	−	Initially, partial recovery of vision and motor control, then worsening	Absence of clinical improvement; radiological worsening	Absence of improvement	−	Absence of improvement	Improvement after several weeks in 2 relapses, worsening in the third relapse	Initial mild improvement then worsening	−	NM
Biotin dose (mg)	15	10	10	20	60	NM	20	20	20	20	10	15	30	30	20
Disease course	Notable improvement	Regression of pyramidal signs, visual clinical and electrophysiological improvement	Could walk and run, mild residual spasticity, control VA: 6/36	Could walk with assistance, control VA: 20/30 RE, 20/25 LE, VEP unchanged	Could walk without aid	Motor and visual improvement	Dramatic recovery of skin and hair, spasticity remained but could walk with aid, control VA: 20/25	Full motor recovery, residual speech and cognitive impairment, moderate hearing loss	Marked improvement, residual spasticity	Motor improvement (could walk without aid), visual acuity remained unchanged	Complete motor improvement, visual acuity 7/10 in LE, 9/10 in RE	Marked improvement: persistent paraparesis but could walk	Complete improvement, developmental gains	Complete improvement	Marked motor improvement (full ambulation restored), partial visual improvement

Abbreviations: CC, corpus callosum; CMJ, cervicomedullary junction; CNS, central nervous system; CSF, cerebrospinal fluid; F, female; LE, left eye; LL, lower limbs; M, male; MN, motor neuron; MRI, magnetic resonance imaging; NM, not mentioned; ON, optic nerves; PGM, periaqueductal gray matter; RE, right eye; SC, spinal cord; VA, visual acuity; VEP, visual evoked potentials; y, years.

⁣^∗^The exact geographic origin was not specified.

## Data Availability

The data used to support the findings of this study are included within the article.
